# Stress-Induced Antinociception in Fish Reversed by Naloxone

**DOI:** 10.1371/journal.pone.0071175

**Published:** 2013-07-30

**Authors:** Carla Patrícia Bejo Wolkers, Augusto Barbosa Junior, Leda Menescal-de-Oliveira, Anette Hoffmann

**Affiliations:** Department of Physiology, School of Medicine of Ribeirão Preto, University of São Paulo, Ribeirão Preto, São Paulo, Brazil; Cajal Institute, Consejo Superior de Investigaciones Científicas, Spain

## Abstract

Pain perception in non-mammalian vertebrates such as fish is a controversial issue. We demonstrate that, in the fish *Leporinus macrocephalus*, an imposed restraint can modulate the behavioral response to a noxious stimulus, specifically the subcutaneous injection of 3% formaldehyde. In the first experiment, formaldehyde was applied immediately after 3 or 5 min of the restraint. Inhibition of the increase in locomotor activity in response to formaldehyde was observed, which suggests a possible restraint-induced antinociception. In the second experiment, the noxious stimulus was applied 0, 5, 10 and 15 min after the restraint, and both 3 and 5 min of restraint promoted short-term antinociception of approximately 5 min. In experiments 3 and 4, an intraperitoneal injection of naloxone (30 mg.kg^−1^) was administered 30 min prior to the restraint. The 3- minute restraint-induced antinociception was blocked by pretreatment with naloxone, but the corresponding 5-minute response was not. One possible explanation for this result is that an opioid and a non-preferential μ–opioid and/or non-opioid mechanism participate in this response modulation. Furthermore, we observed that both the 3- and 5- minutes restraint were severely stressful events for the organism, promoting marked increases in serum cortisol levels. These data indicate that the response to a noxious stimulus can be modulated by an environmental stressor in fish, as is the case in mammals. To our knowledge, this study is the first evidence for the existence of an endogenous antinociceptive system that is activated by an acute standardized stress in fish. Additionally, it characterizes the antinociceptive response induced by stress in terms of its time course and the opioid mediation, providing information for understanding the evolution of nociception modulation.

## Introduction

Pain perception in fish is a controversial issue. According to some authors, the nociceptive responses in fish are purely reflex, associating the pain experience with the presence and degree of differentiation of neocortical structures, which are absent in fish [1], [2]. However, studies demonstrate that the fish nervous system has anatomical structures that can sustain complex behavioral responses to noxious stimuli. Teleost fish can perceive and respond to chemical, thermal and electric shock noxious stimuli [3]]–[[5]. There is evidence that fish have nociceptors with characteristics that are similar to those of mammals [6]]–[[9], and present behavioral and physiological responses to noxious stimuli [3]]–[[5], [10], [11]. Experimental evidence also indicates that fish can learn to avoid a noxious stimulus by associating it with a specific area of the tank and that they retain this information, avoiding a return to this area after the stimulus [12], [13]. Furthermore, this avoidance learning is flexible and can be modified according to the intensity of the stimulus and the situation [13].

A functional opioid system was also observed in teleost fish, which includes the presence of opioid receptors similar to those of mammals [14], [15]. Enkephalin-like substances are present in various brain regions of goldfish [16], [17], catfish [16] and rainbow trout [18]. In addition, systemic pretreatment with morphine has a dose dependent antinociceptive effect [19] and reverses the respiratory and behavioral responses induced by noxious stimuli [3], [10]; the treatment with tramadol also increases the nociceptive threshold [5] suggesting the existence of an antinociceptive opioid system in fish. Furthermore, the inhibition of a nociceptive response was described in rainbow trout that were submitted to social subordination, a non-standardized chronic stress [20]. However, the opioidergic modulation of this endogenous antinociception has not been demonstrated. In piauçu the presence of conspecific alarm substance also promotes endogenous antinociception and this response can be blocked by naloxone, suggesting an opioidergic modulation of the antinociception [21].

Despite these evidences, the participation of the opioid system in the endogenous antinociception induced by a standard acute stress and the time course of this response in fish have not been evaluated. Thus, the purpose of this study was to evaluate whether short-term restraint, a standard stressor, can promote the activation of the antinociceptive system in the piauçu fish *Leporinus macrocephalus* as well as the time course and the participation of the opioid system in this response, using naloxone, a preferential µ-opioid receptor antagonist.

## Materials and Methods

### Husbandry and set-up

A total of 172 juvenile piauçu (*Leporinus macrocephalus*) (17.56±5.6 g weight), that were immature and two months old, were used. The animals were obtained from a fish farm and were kept in stock tanks (100×100×60 cm; n = 50) until the experiments. Five days before the experiments, they were transferred to individual glass aquaria (40×22×20 cm, ∼18L) in a closed system with aerated water (pH: 7.39±0.06; temperature: 26°C±1°C; unionized ammonia (NH_3_): lower than 0.04 mg.L^−^^1^) (Figure S4). During the experiment the water was not replaced to avoid disturbance. The side walls of the aquaria were covered with opaque white paper to isolate fish from visual stimuli of conspecifics in neighboring aquaria and from the experimenter. The animals were subjected to a light/dark cycle of 12:12 h (start 07:00 and end 19:00) and fed daily with pelleted food for fish (PURINE), corresponding to 3% of their biomass. Feeding was stopped 24 hours before the experiments. All of the experiments were conducted at the same time of day (between 8:00 and 10:00 a.m.) to avoid circadian interference.

### Drugs

Formaldehyde (Formaldehyde P.A. –A.C.S. 37%, pKa = 13.3, stabilized with 10% methanol, Merck, Darmstadt, FRG) was diluted in sterile saline. Approximately 20-µl of volume of 3% formaldehyde was applied subcutaneously using a 1-ml syringe and a 22 G×1’ needle (BD, Franklin Lakes, USA). Naloxone hydrochloride (Tocris Bioscience, Bristol, UK) was diluted in sterile saline and injected intraperitoneally (30 mg/kg, 0.1 ml/10 g fish weight) using the same type of syringe and needle.

### Nociceptive test

Subcutaneous injections of 3% formaldehyde in the region of the adipose fin were used as the noxious stimulus, reproducing the nociceptive models proposed for rats [22] and adapted for the fish in our laboratory [23] (Figure S5). Formaldehyde is a chemical stimulus that is extensively used to induce nociception in mammals; it promotes behavioral responses by the activation of nociceptors directly in the initial phase and indirectly in the subsequent inflammatory phase [24]. The animals were removed from the water with nylon nets, wrapped in wet cloth for formaldehyde injection, and immediately returned to the water. The procedure was performed without anesthesia to permit the evaluation of the first nociceptive responses and was as brief as possible (approximately 5 seconds) to avoid suffocation. Previous studies from our laboratory have demonstrated that the subcutaneous injection of formaldehyde, but not saline, promotes a marked increase in locomotor activity, principally in the first 5 minutes after the injection [21]; thus, alterations in locomotor activity in this period were used as a nociceptive indicator.

### Restraint stress and cortisol analysis

To restrain the fish, a rectangular metal screen was introduced into the tank (30×20×3 cm), pushing the fish against one wall and preventing its movements for 3 or 5 minutes (depending on the experimental group), without restricting the opercular movements (Figure S4). During the restraint, the experimenter remained behind the aquarium so that the fish could not see him. Serum cortisol levels were analyzed to confirm the efficacy of this procedure in inducing stress. For this purpose, 24 animals were divided into 3 groups: a control group (n = 8), which was not submitted to restraint; a group submitted to 3 min of restraint (n = 8); and a group submitted to 5 min of restraint (n = 8). Five minutes after the restraint, the fish were anesthetized (Buffered MS-222, methanesulfonate tricaine, 0.2 g/l, Sigma, St. Lois, USA), and their blood was sampled from the caudal vein with a non-heparinized sterile syringe. After obtaining the blood sample, the fish were killed by immersion in anesthetic solution (Buffered MS-222, 0.8 g/l). The blood was centrifuged (3000 rpm for 10 minutes at 4°C), and the serum obtained was used to analyze the cortisol concentration by radioimmunoassay (Coat-A-Count Cortisol, DPC, Los Angeles, USA) in an external laboratory. The experimenter was blind to the treatment during the analysis.

### Behavioral analysis

A camera (Sony CCD-TRV 318) coupled to a computer with image capture software (Virtual Dub 1.6.16) was placed in front of the longest face of the aquarium to record the entire experiment (Figure S4). Locomotor activity was evaluated by examining the recording. For quantitative analysis, the distance travelled and the swimming speed during the evaluation time (5 min of baseline and 5 min post-stimulus) were considered. These variables were analyzed with EthoVision XT 7.1 software (Noldus Information Technology, Wageningen, NL), and the data were expressed as the difference (Δ) of the values after (post-stimulus) and before (baseline) methodological interventions (Δ =  post-stimulus–baseline). The experimenter was blind to the treatment during the analysis, and a reliability test for the video analysis was performed.

To ensure that the blockade of the locomotor responses elicited by noxious stimulus after restraint observed in this study were promoted by the mobilization of an endogenous antinociceptive system and not by the stress itself, a sham group was performed to evaluate the influence of the restraint in the basal locomotor activity. For this purpose, 18 animals were divided into 3 groups: a control group (n = 6), which was not submitted to restraint; a group submitted to 3 min of restraint (n = 6); and a group submitted to 5 min of restraint (n = 6). Baseline behavior was recorded (5 min), and the animals were immediately restrained. The fish’s behaviors were again recorded for 5 minutes (post-stimulus).

### Experimental protocol

#### Experiment 1: Influence of the restraint on the nociceptive response

In this experiment, we evaluated the influence of the duration of restraint on the locomotor responses induced by the formaldehyde. For this purpose, 48 fish were randomly divided into 6 groups: saline (SAL, n = 8), formaldehyde (FOR, n = 8), 3 min of restraint + saline (RES(3) + SAL, n = 8), 3 min of restraint + formaldehyde (RES (3) + FOR, n = 8), 5 min of restraint + saline (RES (5) + SAL, n = 8) and 5 min of restraint + formaldehyde (RES (5) + FOR, n = 8). In the beginning of the experiment, the locomotor activity was recorded for 5 min (baseline). Subsequently, the animals were or were not submitted to restraint (3 or 5 min), depending on the experimental group. Subcutaneous injection of saline or 3% formaldehyde was applied and the locomotor activity was immediately recorded for 5 min (post-stimulus) (Figure S1).

#### Experiment 2: Time course of the inhibition of the nociceptive response induced by restraint

In this experiment, the time course of the restraint’s effect on the locomotor response to formaldehyde was evaluated. For this purpose, 60 fish were randomly divided into 8 groups: 0 min (n = 8), 5 min (n = 8), 10 min (n = 8) or 15 min (n = 7) after 3 min of restraint and 0 min (n = 8), 5 min (n = 7), 10 min (n = 7) or 15 min (n = 7) after 5 min of restraint. In the beginning of the experiment, locomotor activity was recorded for 5 min (baseline). Subsequently, the animals were submitted to 3 or 5 min of restraint, depending on the experimental group. A subcutaneous injection of 3% formaldehyde was applied immediately (0 min), 5, 10 or 15 min after the end of restraint, and the locomotor activity was recorded for 5 min (post-stimulus). All experimental groups were compared to the FOR group (fish submitted to formaldehyde subcutaneous injection without restraint–Experiment 1) (Figure S2).

#### Experiment 3: Influence of naloxone pre-treatment on the inhibition of the nociceptive response induced by 3 min of restraint

In this experiment, the effect of naloxone (30 mg.kg^−1^) on the response to formaldehyde after 3 min of restraint was evaluated. For this purpose, 32 fish were randomly divided into 4 groups: saline +3 min of restraint + saline (SAL + RES (3) + SAL, n = 8), saline +3 min of restraint + formaldehyde (SAL + RES (3) + FOR, n = 8), naloxone +3 min of restraint + saline (NAL + RES (3) + SAL, n = 8) and naloxone +3 min of restraint + formaldehyde (NAL + RES (3) + FOR, n = 8). Locomotor activity was recorded for 5 min (baseline) before the intraperitoneal injection of saline or naloxone, depending of the experimental group. After 30 min, the fish were submitted to 3 min of restraint, followed by the subcutaneous injection of saline or 3% formaldehyde and the locomotor activity was recorded for 5 min (post-stimulus) (Figure S3).

#### Experiment 4: Influence of naloxone pre-treatment on the inhibition of the nociceptive response induced by 5 min of restraint

In this experiment, the effect of naloxone (30 mg.kg^−1^) on the response to formaldehyde after 5 min of restraint was evaluated. For this purpose, 32 fish were randomly divided into 4 groups: saline +5 min of restraint + saline (SAL + RES (5) + SAL, n = 8), saline +5 min of restraint + formaldehyde (SAL + RES (5) + FOR, n = 8), naloxone +5 min of restraint + saline (NAL + RES (5) + SAL, n = 8) and naloxone +5 min of restraint + formaldehyde (NAL + RES (5) + FOR, n = 8). Locomotor activity was recorded for 5 min (baseline) before the intraperitoneal injection of saline or naloxone, depending of the experimental group. After 30 min, the fish were submitted to 5 min of restraint, followed by the subcutaneous injection of saline or 3% formaldehyde, and the locomotor activity was recorded for 5 min (post-stimulus) (Figure S3).

### Statistical analysis

The cortisol data were not normally distributed; therefore, a log10 transformation was performed. After transformation, the data presented a normal distribution (Kolmogorov–Smirnov test, P>0.05) and homogeneity of variance (Levene’s test, P>0.05); therefore a one-way ANOVA followed by a post-hoc Tukey’s test (P<0.05) were performed to compare the cortisol levels between the experimental groups.

The locomotor activity (swimming speed and distance travelled) in experiments 1, 2, 3 and 4 and in the sham group presented a normal distribution (Kolmogorov–Smirnov test, P>0.05) and homogeneity of variance (Levene’s test, P>0.05); therefore a one-way ANOVA was performed to compare the locomotor activity between the experimental groups in each experiment. When significant main effects were identified, post-hoc Tukey’s tests (P<0.05) were used to compare the locomotor activity between the experimental groups. In experiment 2, a two-way ANOVA was also performed to evaluate the effects of the duration of the restraint over time (0, 5, 10 and 15 min).

### Ethics statement

This research was conducted in accordance with the Ethical Principles in Animal Research adopted by the National Council for the Control of Animal Experimentation - Brazil (CONCEA–Conselho Nacional de Controle de Experimentação Animal–Brazil) and was approved by the Ethical Committee for Animal Research from the School of Medicine of Ribeirão Preto (FMRP-USP) (Case No. 052/2010).

## Results

There was a significant effect of the restraint on the serum cortisol levels (ANOVA, F _2,21_  = 43.98, P<0.001). A significant increase in the serum cortisol level was observed after 3 (115.87±51.45 ng.mL^−1^) and 5 min (132.12±26.33 ng.mL^−1^) of restraint when compared with fish that were not subjected to this stimulus (8.98±2.34 ng.mL^−1^) (Tukey, P<0.001). There was no difference between the two experimental groups. The basal locomotor activity (Distance: ***Control***–110.61±125.83 cm; ***3*** ***min***–184.03±124.28 cm; ***5*** ***min***–90.90±46.15 cm; and Swimming Speed: ***Control***–0.39±0.41cm.s^−^^1^; ***3*** ***min***–0.61±0.39 cm.s¯^1^; ***5*** ***min***–0.30±0.15 cm.s^−^^1^) was not affected by the restraint (ANOVA, F_2,15_ = 1.820, P = 0.196).

### Experiment 1: Influence of the restraint on the nociceptive response

**Figure 1:**
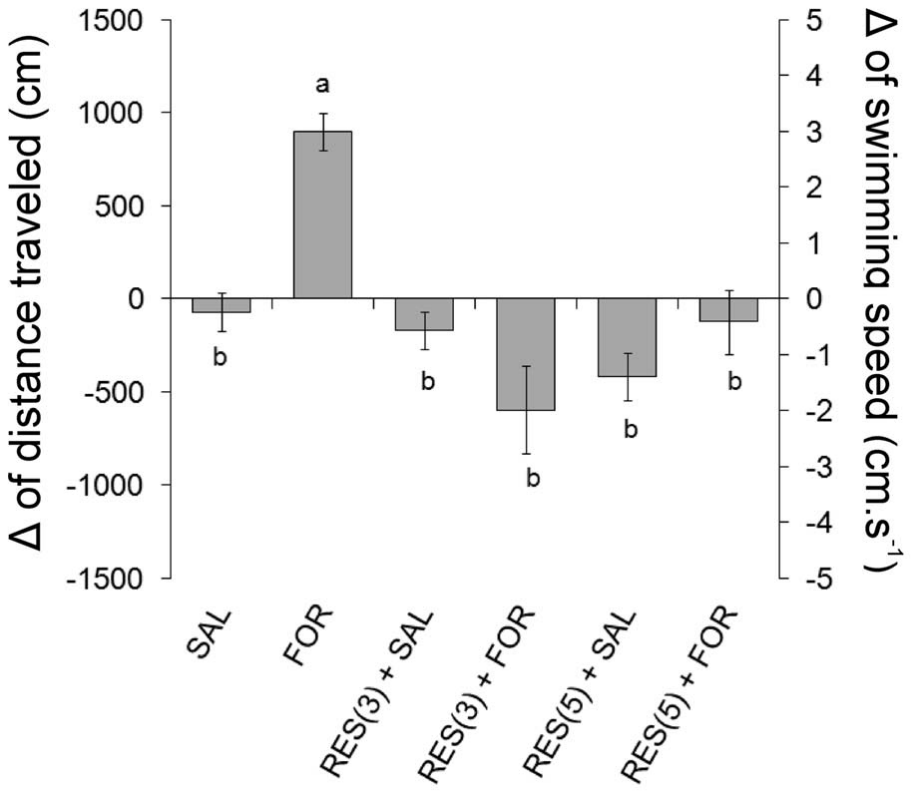
Locomotor activity of *L. macrocephalus* subjected to restraint for 3 and 5 min, followed by subcutaneous injection of 3% formaldehyde. Data are presented as the difference (Δ) between post-stimulus and baseline. Experimental groups: saline (SAL, n = 8), formaldehyde (FOR, n = 8), 3 min of restraint + saline (RES (3) + SAL, n = 8), 3 min of restraint + formaldehyde (RES (3) + FOR, n = 8), 5 min of restraint + saline (RES (5) + SAL, n = 8) and 5 min of restraint + formaldehyde (RES (5) + FOR, n = 8). Different letters indicate significant difference (Tukey test, P<0.05).

There was a significant effect of the restraint on the locomotor response induced by the subcutaneous injection of formaldehyde (ANOVA, F_5,42_  = 12.37, P<0.001). The 3% formaldehyde subcutaneous injection (FOR) induced an erratic pattern of swimming that begins immediately after the drug administration. Significant increases in the distance travelled and swimming speed were observed after formaldehyde injection compared to saline injection (SAL) (Tukey, P<0.001). In animals that were subjected to 3 min of restraint (RES (3) + FOR), the distance travelled and swimming speed values were significantly lower than the values that were observed in unstressed animals (FOR) (Tukey, P<0.001), but were not significantly different from SAL and RES (3) + SAL. The animals that were subjected to 5 min of restraint (RES (5) + FOR) showed behavior patterns similar to those subjected to 3 min of restraint, and the distance travelled and swimming speed values were significantly lower than the values observed in unstressed animals (FOR) (Tukey, P<0.001), but were not significantly different from SAL and RES (5) + SAL. There were no significant differences in the locomotor activity between the animals that were submitted to 3 or 5 min of restraint prior to formaldehyde subcutaneous injection (Figure 1).

### Experiment 2: Time course of the inhibition of the nociceptive response induced by restraint

**Figure 2:**
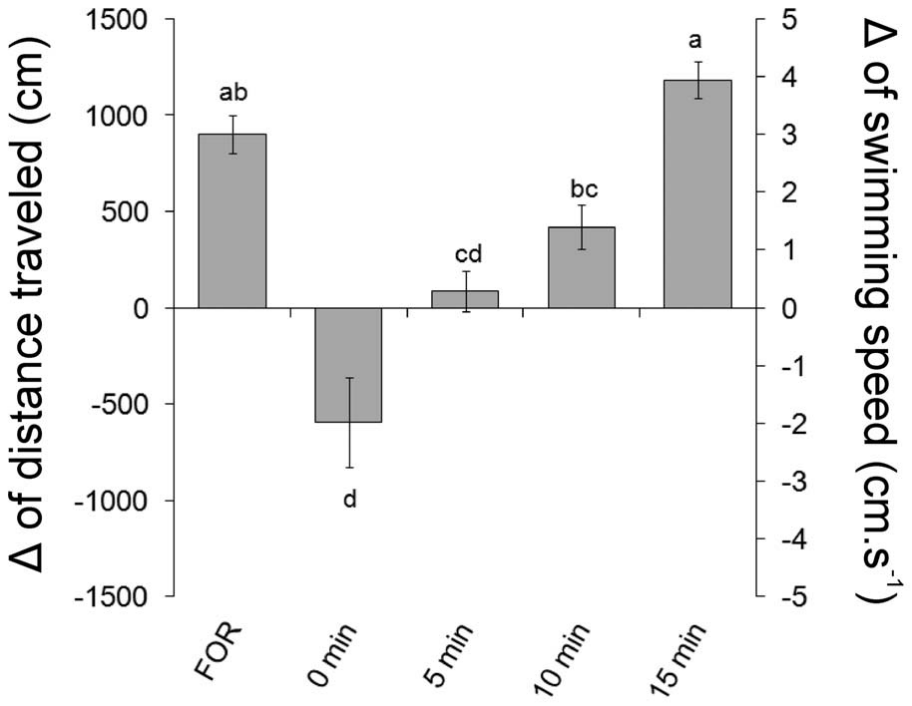
Time course of the effect of 3 min of restraint on the locomotor activity of *L. macrocephalus* subjected to subcutaneous injection of 3% formaldehyde. Data are presented as the difference (Δ) between post-stimulus and baseline. Experimental groups: subcutaneous injection of formaldehyde without restraint (FOR) and applied immediately (0 min, n = 8), 5 (n = 7), 10 (n = 7) or 15 (n = 7) min after the restraint. Different letters indicate significant difference (Tukey test, P<0.05).

There was a significant effect of the time on the inhibition of the locomotor response to formaldehyde induced by 3 min of restraint (ANOVA one-way, F_4,34_ 16.95, P<0.001). The distance travelled and the swimming speed values were significantly lower than those presented by non-immobilized animals when formaldehyde was applied immediately after restraint (0 min) and 5 min after restraint (Tukey, P<0.001). The formaldehyde injections 10 and 15 min after the restraint promoted increases in locomotor activity similar to those presented by non-immobilized animals (Figure 2).

The two-way ANOVA analysis demonstrated no significant effect of the duration of the restraint and the time of injection×duration of restraint interaction on the locomotor response to formaldehyde (F_1,54_ 0.029, P = 0,865 and F_3,54_ 2.06, P = 0.116, respectively). A significant effect was observed for the time of injection on the locomotor response to formaldehyde (F_3,54_  = 18.255, P<0,001).

**Figure 3:**
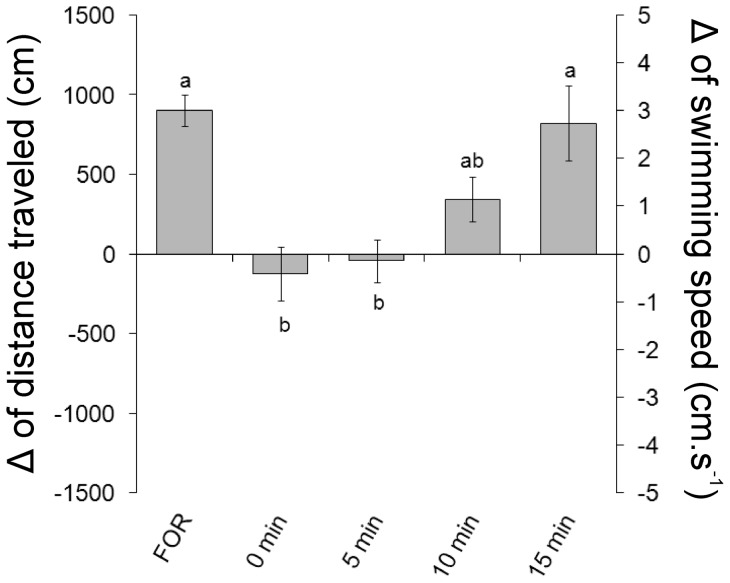
Time course of the effect of 5 min of restraint on the locomotor activity of *L. macrocephalus* subjected to subcutaneous injection of 3% formaldehyde. Data are presented as the difference (Δ) between post-stimulus and baseline. Experimental groups: subcutaneous injection of formaldehyde without restraint (FOR) and applied immediately (0 min, n = 8), 5 (n = 8), 10 (n = 8) or 15 (n = 7) min after the restraint. Different letters indicate significant difference (Tukey test, P<0.05).

There was a significant effect of the time on the inhibition of the locomotor response to formaldehyde induced by 5 min of restraint (ANOVA one-way, F_4,32_  = 6.71, P<0.001). The distance travelled and swimming speed were significantly lower than those presented by non-immobilized animals when formaldehyde was applied immediately after the restraint (0 min) and 5 min after the restraint (Tukey, P<0.001). The formaldehyde injections 10 and 15 min after the restraint promoted increases in locomotor activity, and the distance and swimming speed were similar to those presented by non-immobilized animals (Figure 3).

### Experiment 3: Influence of naloxone pre-treatment on the inhibition of the nociceptive response induced by 3 min of restraint

**Figure 4:**
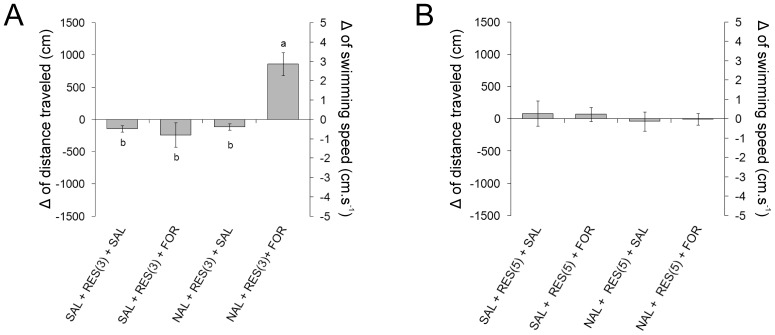
Locomotor activity of *L. macrocephalus* subjected to intraperitoneal injection of naloxone (30 mg.kg^−1^) 30 min before restraint, followed by subcutaneous injection of 3% formaldehyde. Data are presented as the difference (Δ) between post-stimulus and baseline. **A**. 3 min of restraint; **B**. 5 min of restraint. Experimental groups: saline 3 or 5 min of restraint + saline (SAL + RES (3 or 5) + SAL, n = 8), saline 3 or 5 min of restraint + formaldehyde (SAL + RES (3 or 5) + FOR, n = 8), naloxone 3 or 5 min of restraint + saline (NAL + RES (3 or 5) + SAL, n = 8) and naloxone 3 or 5 min of restraint + formaldehyde (NAL + RES (3 or 5) + FOR, n = 8). Different letters indicate significant difference (Tukey test, P<0.05).

A significant effect of the naloxone intraperitoneal injection (30 mg.kg^−^^1^) on the inhibition of the nociceptive response induced by 3 min of restraint was observed (ANOVA, F_3,28_  = 14.65, P<0.001). The naloxone injection 30 min before 3 min of restraint blocked the restraint-induced inhibition of the locomotor response to formaldehyde. In the NAL + RES (3) + FOR group (naloxone-treated animals before the restraint followed by formaldehyde subcutaneous injection), the distance and speed values were significantly higher than those presented by SAL + RES (3) + SAL, SAL + RES (3) + FOR and NAL + RES (3) + SAL (Tukey, P<0.001) (Figure 4 A).

### Experiment 4: Influence of naloxone pre-treatment on inhibition of the nociceptive response induced by 5 min of restraint

No effect of the naloxone intraperitoneal injection (30 mg.kg^−^^1^) on the inhibition of the nociceptive response induced by 5 min of restraint was observed (ANOVA, F_3,28_  = 0.169, P  = 0.916). In the NAL + RES (5) + FOR group (naloxone-treated animals before the restraint followed by formaldehyde subcutaneous injection), the distance traveled and the speed values were similar to observed in the SAL + RES (5) + SAL, SAL + RES (5) + FOR and NAL + RES (5) + SAL groups (Figure 4 B).

## Discussion

Our findings demonstrate an endogenous opioid antinociceptive system, that is activated by an acute stress, modulating the response to formaldehyde in fish, similar to that observed in mammals. This important evidence of nociception modulation, associated to previous studies in the field [20], [21], provides new perspectives on the evolution of nociception in vertebrate phylum.

We used subcutaneous injections of 3% formaldehyde in the adipose fin region to induce nociceptive-related behavior, a technique developed and currently used in our laboratory for piauçu fish [21], [23]. The application of formaldehyde promotes an immediate and intense increase in locomotor activity, associated with an erratic swim pattern, possibly indicating the perception of the noxious stimulus. An increase in locomotor activity was also associated with nociception in fish using chemical (formaldehyde subcutaneous injection) [21] and mechanical (tailfin clipping) [25] noxious stimuli. Electrophysiological evidence of the activation of nociceptors by chemical noxious substances has also been shown [26], [27]. Beyond the locomotor arousal, other behavioral alterations are related to the application of a chemical noxious stimulus in fish, including an increase in the respiratory rate [28] and performance of atypical behaviors such as “rubbing” and “rocking” [3], [28]. The variety of nociceptive-related behaviors described in various fish species indicates that the behavioral and physiological responses to a nociceptive event are species-specific among fish [28], [29], [30], [31].

The behavioral response to formaldehyde is inhibited by previous restraint, which suggests that an antinociceptive effect is induced by this stressor. Although restraint stress was highly stressful for the fish, promoting a marked increase in the cortisol levels, the suppression of the locomotor response to formaldehyde cannot be attributed to an effect of the stress and/or cortisol in the activity of the fish, because no such effect was observed after submitting the fish to only the restraint. Thus, the antinociception observed could indicate the activation of a stress-induced endogenous antinociceptive system. In mammals, the endogenous antinociceptive system is a component of defensive behavior and can be mobilized in stress situations or during encounters where there is a risk of injury to the animal [32]. The existence of an endogenous antinociceptive system was previously demonstrated in rainbow trout [20] and piauçu [21]. The existence of an endonegous antinociceptive system in fish suggests that this system evolved early in the vertebrate phylum because it is present in a basal vertebrate group, the fish.

The existence of an endogenous antinociceptive system induced by restraint has been described in mammals and amphibians [33]]–[[37]. Its activation promotes the inhibition of nociceptive responses, as evaluated by the tail flick, hot plate, acetic acid and formaldehyde tests [33]]–[[37]. In our study, a short-term restraint was sufficient to produce antinociception in fish. Although studies with mammals and amphibians use a longer period of restraint to induce antinociception (0.5 to 4 hours) [33]]–[[37], another study in mammals showed that this effect can also be observed a few minutes (5 min) after the start of the restraint [38].

In addition to presenting a rapid activation, the restraint promotes short-term antinociception in fish that lasts approximately 5 min. Studies on mammals show that the duration of the antinociception is variable depending on the type and intensity of the stress. In mammals, short-term antinociception was described in studies that used forced swimming [39], immobilized-water immersion [40], and various parameters of footshock [40],[41], with durations of 5 to 15 min. Long-term antinociception was promoted by a long period of restraint (30 min) [33] with a duration of more than 60 min. The short-term antinociception observed in the present study could be related to the short period of restraint that was used in the experimental protocol. Longer periods of restraint could promote antinociception for a different duration.

By analyzing the participation of the µ-opioid receptor on the restraint -induced antinociception, using naloxone, we observed that this response seems to be mediated by distinct mechanisms in fish, depending on the duration of the stress. The antinociception promoted by 3 min of stress is blocked by naloxone, which suggests the participation of a preferential μ-opioid mechanism, while the 5 min antinociception, not blocked by naloxone, suggests the participation of non-preferential μ-opioid and/or non-opioid mechanisms. These results corroborate studies on mammals, which also show that stress-induced antinociception can be blocked or not by naloxone, depending on temporal and spatial factors [40]]–[[47].

The activation of different mechanisms of nociceptive modulation in fish supports the idea that the process underlying nociception and antinociception in this group can be complex. There is evidence for the existence of a functional opioid system, with the presence of the opioid receptor µ similar to mammalian receptors [15] and the inhibition of nociceptive-related behaviors by morphine and tramadol application [3], [5]. The activation of an opioid endogenous analgesic system was described in piauçu that were submitted to the conspecific alarm substance [21]. Furthermore, in rainbow trout, the stress promoted by one week of social subordination can activate an endogenous antinociceptive system [20]. However, this study did not evaluate the participation of the opioid system in this response. To our knowledge, the present study is the first to demonstrate the modulation of nociceptive behaviors by an endogenous opioid system in fish activated by a standard acute stressful stimulus. Furthermore, our results suggest possible involvement of other systems of nociceptive processing, which are non-preferential μ-opioid and/or non-opioid. Studies of mammals have shown that stress-induced antinociception can mobilize other neurochemical systems, such as the serotonergic, noradrenergic, GABAergic and endocannabinoid [32], [48]]–[[51]. Thus, further studies are required to assess the nature of stress-induced antinociception from 5 min of restraint in fish. Ongoing studies in our laboratory suggest the involvement of endocannabinoid, serotonergic and noradrenergic systems in this antinociceptive response.

In summary, the results of this study, which show the activation of an endogenous antinociceptive system by stress in fish, provide important evidence for the existence of an endogenous modulation of nociception in this vertebrate group. From a phylogenetic perspective, these data are an important tool for elucidating the evolution of nociception in the vertebrate phylum. Our results, associated to previous studies in the field [20], [21], also show that the endogenous antinociceptive system, related to the modulation of nociception, evolved early in vertebrate history because it is present in a basal vertebrate group, fish. Furthermore, these results, together with previous studies in this field, are relevant to animal welfare and the ethics involved in the use of fish in research and commercial activity.

## Supporting Information

Figure S1 **Schematic drawing of the experimental sequence of the experiment 1.** B–Baseline recording; S–Saline subcutaneous injection; F–Formaldehyde subcutaneous injection; R–Restraint; PS–Post-stimulus recording.
(TIF)

Figure S2 **Schematic drawing of the experimental sequence of the experiment 2.** B–Baseline recording; R–Restraint; F–Formaldehyde subcutaneous injection; PS–Post-stimulus recording.
(TIF)

Figure S3 **Schematic drawing of the experimental sequence of the experiments 3 and 4.** B–Baseline recording; S–Saline intraperitoneal injection; N–Naloxone intraperitoneal injection; R–Restraint; S–Saline subcutaneous injection; F–Formaldehyde subcutaneous injection; PS–Post-stimulus recording.
(TIF)

Figure S4 **Experimental set-up. A.** Position of the camera in relation to the aquaria; **B.** Experimental aquarium; **C.** Metal screen used to restrain the fish; **D.** Experimenter restraining the fish.
(TIF)

Figure S5 **Localization of the subcutaneous injection of 3% formaldehyde in the region of the adipose fin.**
(TIF)
