# Supplementary figures and images for: Stress-Induced Antinociception in Fish Reversed by Naloxone
Source: PLoS One. 2013 Jul 30;8(7):e71175. doi: 10.1371/journal.pone.0071175 (PMC3728202; doi:10.1371/journal.pone.0071175)

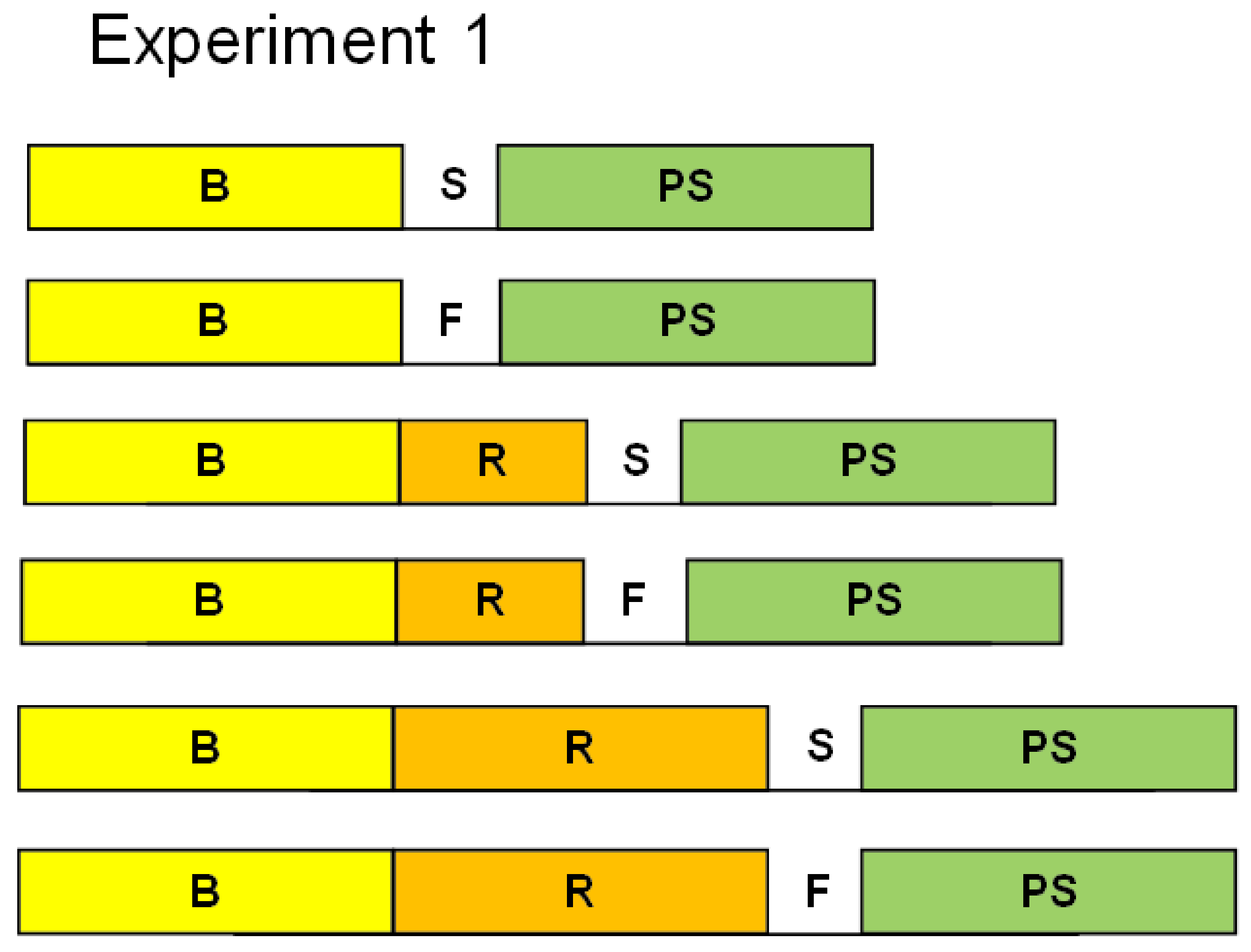

Supplement: Figure S1 — Schematic drawing of the experimental sequence of the experiment 1. B–Baseline recording; S–Saline subcutaneous injection; F–Formaldehyde subcutaneous injection; R–Restraint; PS–Post-stimulus recording. (TIF) [file pone.0071175.s001.tif]

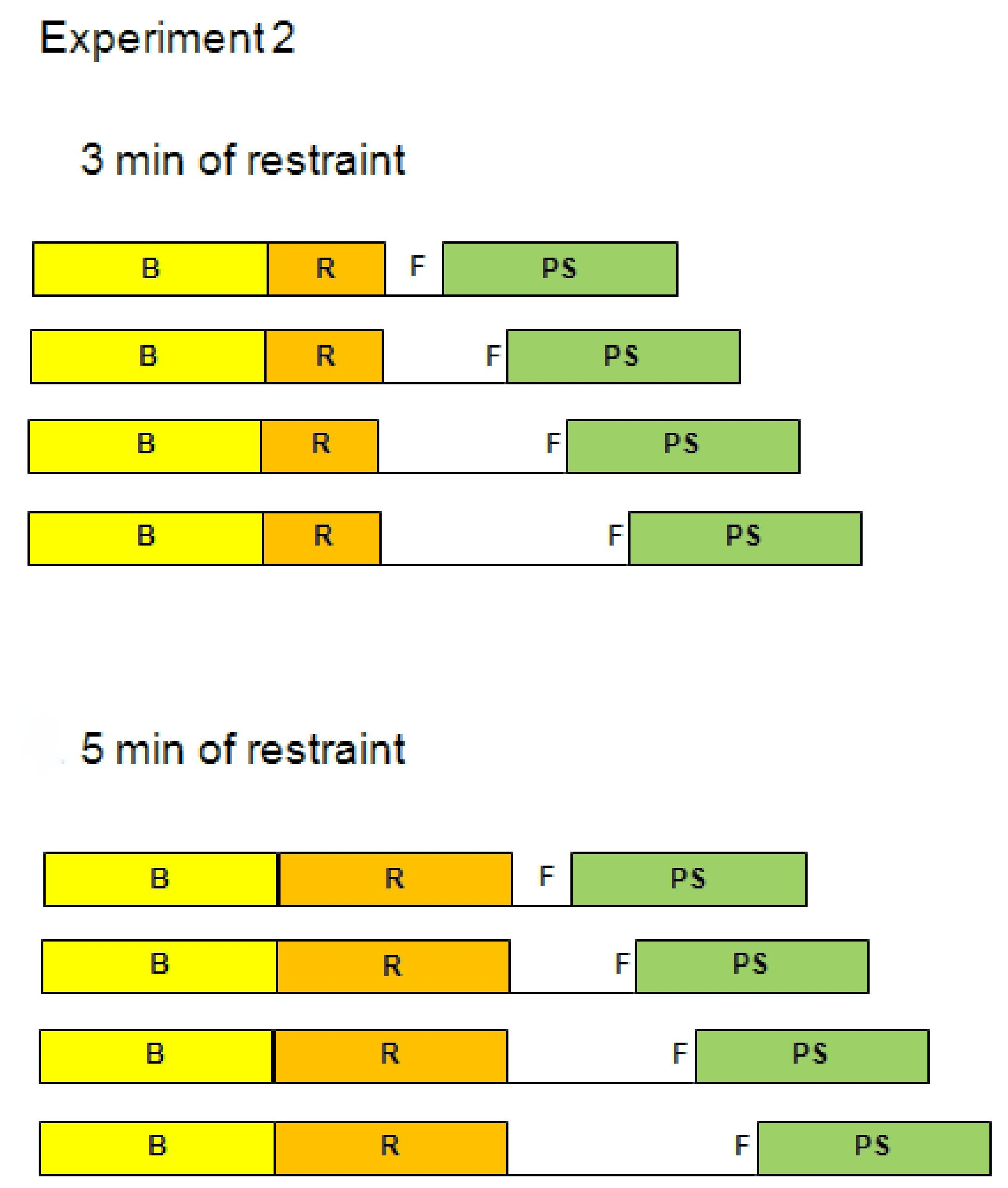

Supplement: Figure S2 — Schematic drawing of the experimental sequence of the experiment 2. B–Baseline recording; R–Restraint; F–Formaldehyde subcutaneous injection; PS–Post-stimulus recording. (TIF) [file pone.0071175.s002.tif]

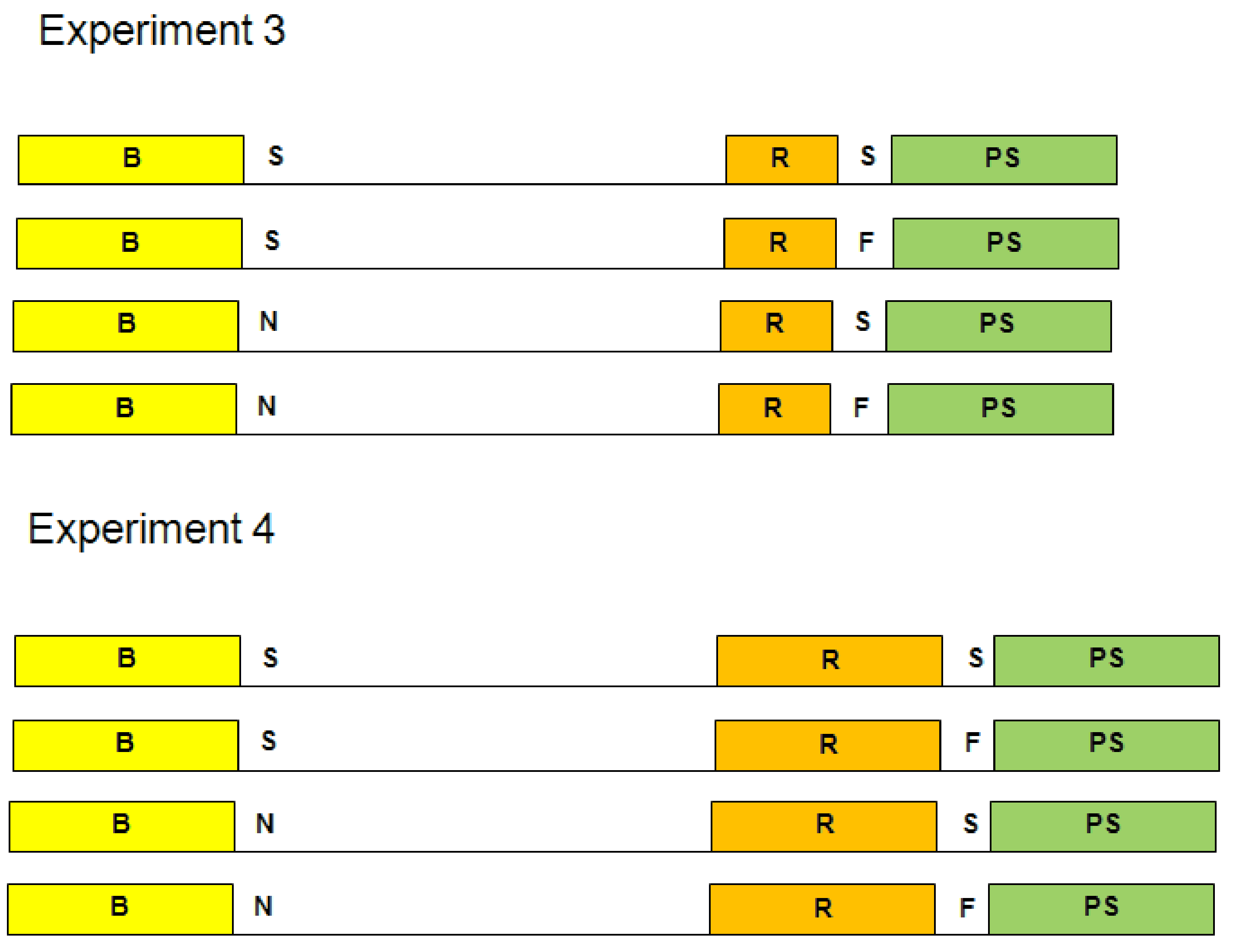

Supplement: Figure S3 — Schematic drawing of the experimental sequence of the experiments 3 and 4. B–Baseline recording; S–Saline intraperitoneal injection; N–Naloxone intraperitoneal injection; R–Restraint; S–Saline subcutaneous injection; F–Formaldehyde subcutaneous injection; PS–Post-stimulus recording. (TIF) [file pone.0071175.s003.tif]

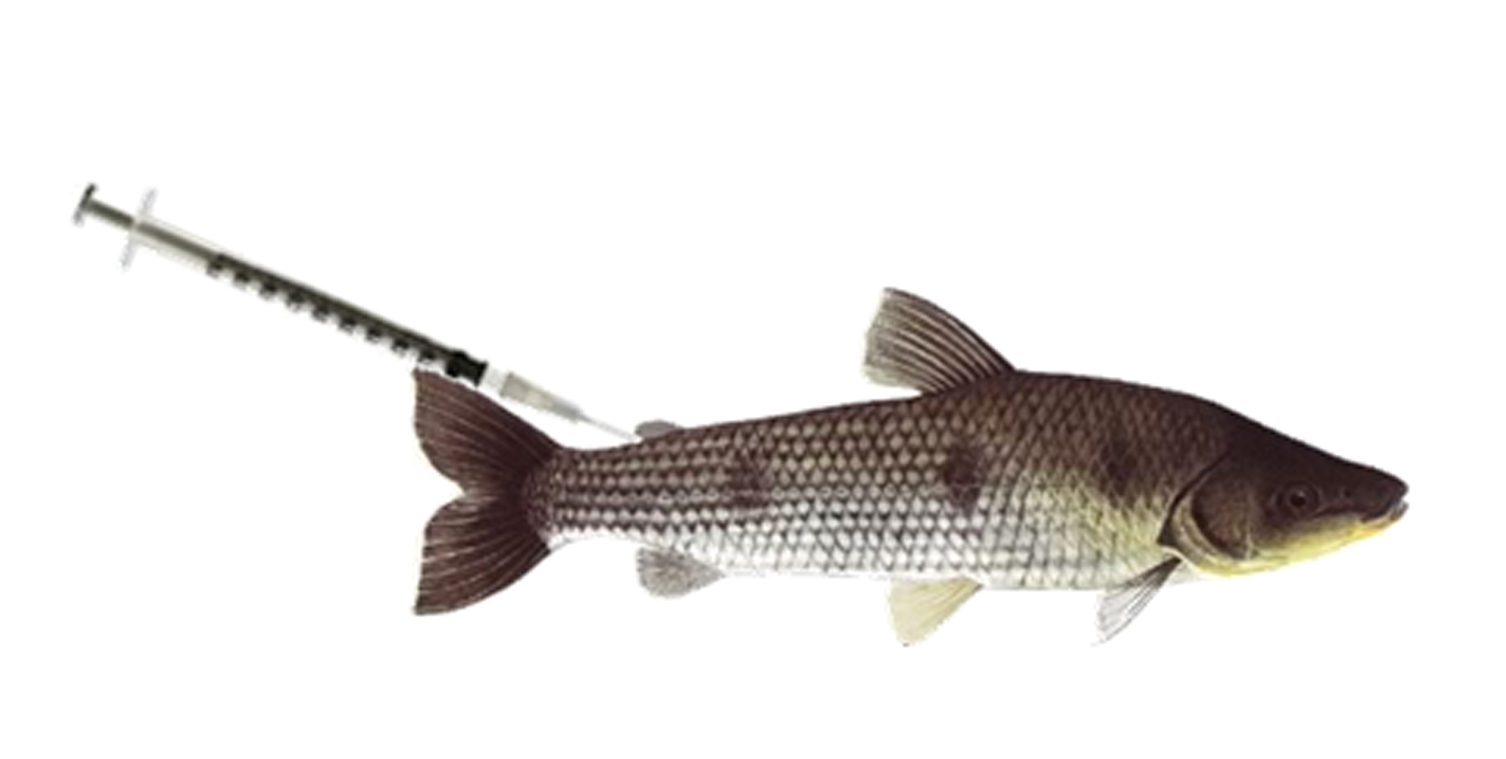

Supplement: Figure S5 — Localization of the subcutaneous injection of 3% formaldehyde in the region of the adipose fin. (TIF) [file pone.0071175.s005.tif]
